# Does cognitive aid app design influence the speed of actions during a critical event?: A simulation study

**DOI:** 10.1111/pan.15037

**Published:** 2024-11-07

**Authors:** Brady Still, Anna Clebone

**Affiliations:** ^1^ University of Chicago Chicago Illinois USA

**Keywords:** anesthesia safety, checklists, cognitive aids, emergency manual, pedi crisis app

## 1 INTRODUCTION

**FIGURE 1:**
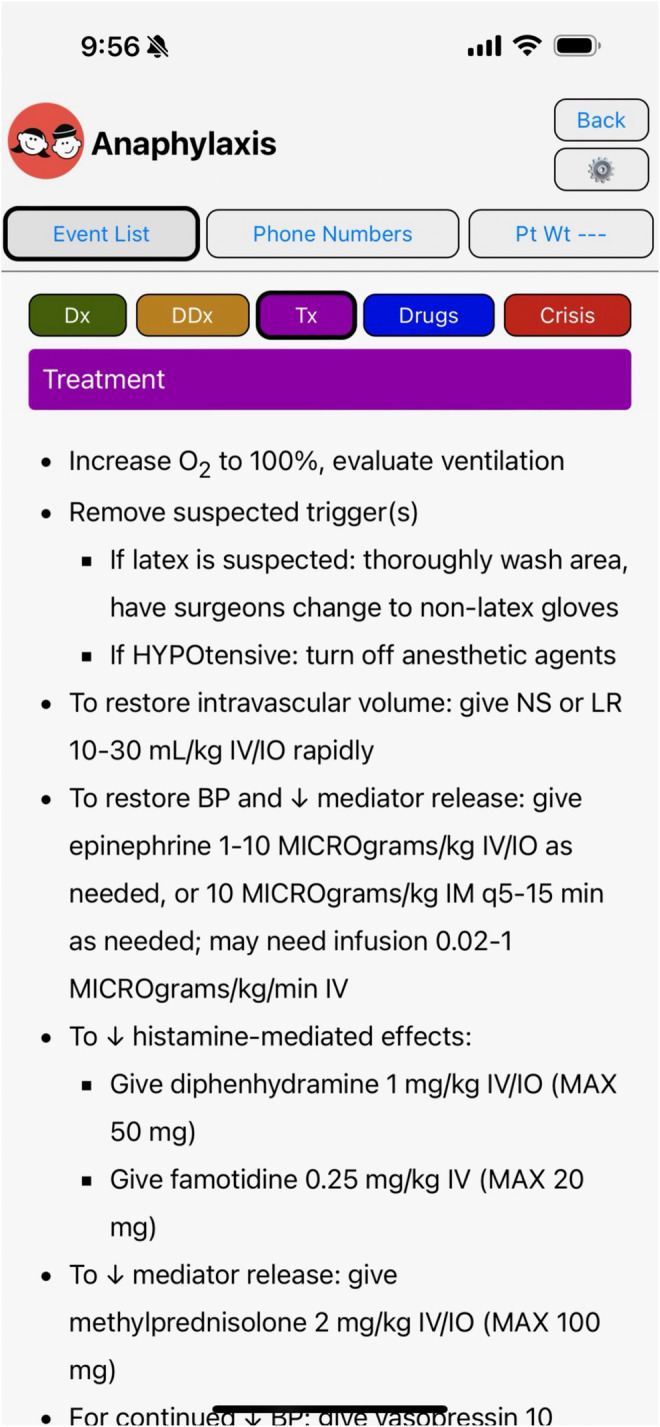
Pedi Crisis 2.0 app from the Society for Pediatric Anesthesia. Available free from the Society for iPhone and Android at: https://pedsanesthesia.org/pedi‐crisis‐app/.

Deliberately designed physical critical event cognitive aids facilitate faster information acquisition.^1^, ^2^ It is unknown, however, if design is also important for electronic cognitive aids. The Pedi Crisis 2.0 Mobile Application^3^ (Figure 1) was created by the Society for Pediatric Anesthesia to help anesthesiology clinicians find discrete information during critical events. In this app, information is grouped into easily accessible sections corresponding to the most common reasons that clinicians access cognitive aids during critical events: to obtain discrete information such as a drug dose, ascertain additional treatment ideas or differential diagnoses, or corroborate that no necessary actions were missed, a concept known as ‘sampling.’^4^ We hypothesize that this human‐factors informed design for an electronic pediatric critical event app will decrease the time to find critical information.

## 2 METHODS

After the University of Chicago IRB exempt designation, IRB22‐1884, data was collected from a convenience sample of currently practicing anesthesia providers from 12/2022 to 1/2024 with verbal consent. Methods were similar to a previous study,^1^ with several exceptions. Two electronic aids were tested using an Apple iPad Pro, each with identical information: (1) the Pedi Crisis 2.0 app and (2) a control app in list format created using Bubble.io. This control app differed from the Pedi Crisis checklists in pdf/paper format: (https://pedsanesthesia.org/critical‐events‐checklists/). The pdf/paper format Pedi Crisis checklists are optimized according to human factors principles for an 8.5 × 11 sheet of paper, and if viewed on an electronic device, both vertical and horizontal scrolling would likely be required. The bubble.io control app was a simple list of information, and no horizontal and minimal vertical scrolling was required. Demographic and survey data were collected using RedCAP, University of Chicago NIH CTSA UL1TR002389.^5^

Subjects were tested individually, sitting at a desk in an empty conference room by a single author (B.S.). Subjects were asked to examine both types of cognitive aid in a randomized order and then choose one to use for training (bradycardia). After finishing the training scenario, subjects were presented with the individual study scenarios, one at a time. The study scenarios consisted of each of eight possible combinations of the four test scenarios (anaphylaxis, cardiac arrest, bronchospasm, and air embolism) and two cognitive aid designs. To decrease the possibility of a learning effect, the eight permutations were offered in a quasi‐randomized fashion using a revised Latin square design, such that no cognitive aid design type was offered twice in a row.^6^ In the crisis management scenario stem, subjects were informed that they had finished a discrete series of actions and had decided to access the cognitive aid for a specific reason: to look for additional treatments (anaphylaxis), identify causes for the emergency (cardiac arrest), find a correct drug dose (bronchospasm), or look for a differential diagnosis (air embolism). Subsequent to reading each scenario (electronic supplemental material: scenarios) and the assigned task, subjects accessed the corresponding cognitive aid, and a timer was started. The timer was stopped when subjects verbalized that they had found the requested data. Subjects were told to discover the information as fast as possible and knew that they were being timed.^1^

### 2.1 Statistical analysis

The amount of time to find the specified information in each of the two app designs was the primary outcome. A priori, G*Power version 3.1.9.6^7^ was used to determine the needed sample size for the primary outcome. A 2 × 4 repeated‐measures ANOVA showed that a sample size of 8 would be needed, with an effect size *f* = 0.6; power = 0.95; and significance level = 0.05 (without Bonferroni adjustment). The effect size of 0.6 is similar to that used for several previous cognitive aid design evaluation studies.^1^, ^8^

Data were analyzed via the GIGA and Social Science Statistics calculators (web‐based), using methods similar to previous studies that evaluated time to information finding. To test for the normality of distribution, we used the Shapiro–Wilk test. The Wilcoxon Signed‐Rank test was used to analyze the data regarding the overall time required to find the information. All *p* values reported here are unadjusted.

## 3 RESULTS

Five faculty anesthesiologists, two residents, and one fellow completed all study procedures, four male, three female, one declined to answer, with a mean (SD) age of 36 (11.1), and 7.1 (10.5) years of experience. All subjects were familiar with cognitive aids (malignant hyperthermia, local anesthetic systemic toxicity), but only one subject was familiar with the Society for Pediatric Anesthesia Crisis Checklist. Two subjects had previously used a cognitive aid during ten or more critical events, and one subject had previously used an aid during one critical event.

Response times were compiled for each of the two conditions. Subjects found and extracted information more rapidly using the experimental format compared to the control format; median [interquartile range] 6 [4.5–11] seconds versus 10.5 [6–15.5] seconds, *p* = 0.023, *Z* = −2.23, SD = 46.25.

## 4 CONCLUSION

We found that a human‐factors informed cognitive aid mobile application design led to faster acquisition of needed information during a low‐fidelity simulation study of critical events compared to a control design using a list format. Existing data supports the time sensitive nature of critical events. For example, for every minute delay in performing CPR, survival is thought to decrease by 10%.^9^ This study adds to the literature in that it examined electronic cognitive aids, whereas a previous critical event information finding study looked at paper cognitive aids.^1^ Future research should examine cognitive aid design in real‐world conditions.

## FUNDING INFORMATION

The authors have not received funding for the development of Pedi Crisis.

## Supporting information


Data S1.

## Data Availability

Data will be available from the authors upon reasonable request.

## References

[1] Clebone A , Burian BK , Tung A . Matching design to use: a task analysis comparison of three cognitive aid designs used during simulated crisis management. Can J Anaesth. 2019;66(6):658‐671. 30805904 10.1007/s12630-019-01325-8

[2] Frykholm P . Visual aids for pediatric airway management. Paediatr Anaesth. 2020;30(3):371‐374. 31841250 10.1111/pan.13789

[3] Clebone A , Strupp KM , Whitney G , et al. Development and usability testing of the Society for Pediatric Anesthesia Pedi Crisis Mobile Application. Anesth Analg. 2019;129(6):1635‐1644. 31743185 10.1213/ANE.0000000000003935

[4] Clebone A , Watkins SC , Tung A . The timing of cognitive aid access during simulated pediatric intraoperative critical events. Paediatr Anaesth. 2020;30(6):676‐682. 32271972 10.1111/pan.13868

[5] Harris PA , Taylor R , Thielke R , Payne J , Gonzalez N , Conde JG . Research electronic data capture (REDCap)—a metadata‐driven methodology and workflow process for providing translational research informatics support. J Biomed Inform. 2009;42(2):377‐381. 18929686 10.1016/j.jbi.2008.08.010 PMC2700030

[6] Zeelenberg R , Pecher D . A method for simultaneously counterbalancing condition order and assignment of stimulus materials to conditions. Behav Res Methods. 2015;47(1):127‐133. 24903688 10.3758/s13428-014-0476-9

[7] Faul F , Erdfelder E , Lang AG , Buchner A . G*power 3: a flexible statistical power analysis program for the social, behavioral, and biomedical sciences. Behav Res Methods. 2007;39(2):175‐191. 17695343 10.3758/bf03193146

[8] Marshall SD , Sanderson P , McIntosh CA , Kolawole H . The effect of two cognitive aid designs on team functioning during intra‐operative anaphylaxis emergencies: a multi‐centre simulation study. Anaesthesia. 2016;71(4):389‐404. 26792648 10.1111/anae.13332 PMC5066652

[9] Larsen MP , Eisenberg MS , Cummins RO , Hallstrom AP . Predicting survival from out‐of‐hospital cardiac arrest: a graphic model. Ann Emerg Med. 1993;22(11):1652‐1658. 8214853 10.1016/s0196-0644(05)81302-2

