# Supplementary material for: Does cognitive aid app design influence the speed of actions during a critical event?: A simulation study
Source: Paediatr Anaesth. 2024 Nov 7;35(2):175–7. doi: 10.1111/pan.15037 (PMC11701960; doi:10.1111/pan.15037)
Supplement: Supplementary file 1 — Data S1. [file PAN-35-175-s001.docx]

Electronic Supplemental Material: Scenarios

**Scenario Development**

Using Google Analytics, we obtained data on the most frequently viewed event screens in the Pedi Crisis 2.0 App during real-life use. We chose these four events to correspond to the scenarios developed for this research.

***TRAINING BRADYCARDIA SCENARIO***

4 yo male for tonsillectomy and adenoidectomy
PMH: Trisomy 21, transitional AVSD
PSH: AVSD repair
Events: Pre-induction heart rate is 110. During mask induction, the patient became bradycardic to the 60s with concomitant hypotension. You administered atropine and epinephrine; however, the patient entered complete heart block and remains unstable. You decide to transcutaneously pace the patient, but cannot recall the exact steps for doing so. When you access the cognitive aid, I’d like you to find instructions for transcutaneous pacing for bradycardia, tell me as quickly as you can what the first step is for transcutaneous pacing, and where that information can be found on the cognitive aid.

Access the cognitive aid now.

**SCENARIO 1: ANAPHYLAXIS**

8 yo male for right sided inguinal hernia repair
PMH: none
PSH: none
Events: After cefazolin was given, patient had erythema, hives, bronchospasm, and hypotension, then desaturated to an S_p_O_2_ of 92%. You give albuterol, a fluid bolus, and epinephrine, and the blood pressure, S_p_O_2_, and wheezing begin to improve. You suspect that the cause of the symptoms is anaphylaxis. You are drawing a blank on what you want to do next after your initial response. When you access the cognitive aid, I’d like you to look for additional treatments, and I’d like you to tell me as quickly as you can what those additional treatments are and where that information can be found on the cognitive aid.

Access the cognitive aid now.

**SCENARIO 2: CARDIAC ARREST**

10 yo female for exploratory laparotomy following MVC
PMH: none
PSH: none
Events: Shortly after entry into the abdomen, the patient became profoundly hypotensive and tachycardic. Despite aggressive resuscitation, her hemodynamics failed to improve, and she ultimately suffered a bradycardic arrest that degenerated into PEA. PALS is ongoing, but in the heat of the moment, you are struggling to remember the reversible causes of PEA. When you access the cognitive aid, I’d like you to look for reversible causes of cardiac arrest, and I’d like you to tell me as quickly as you can what those additional causes are and where that information can be found on the cognitive aid.

Access the cognitive aid now.

**SCENARIO 3: BRONCHOSPASM**

12 yo male for MRI under GA
PMH: moderate persistent asthma
PSH: none
Events: Over the course of the first 30 minutes of the anesthetic, the peak inspiratory pressures required to achieve adequate minute ventilation gradually increased to 32 mmHg, and the patient’s S_p_O_2_ decreased to 84%. You auscultated the patient’s lung fields and heard bilateral wheezing. You removed the patient from the scanner, and, suspecting bronchospasm, administered albuterol with improvement in the S_p_O_2_ to 89%. You decide to next give ketamine and magnesium, but you forget what doses to give. When you access the cognitive aid, I’d like you to tell me as quickly as you can the dose of ketamine for bronchospasm, and where that information can be found on the cognitive aid.

Access the cognitive aid now.

**SCENARIO 4: AIR EMBOLISM**

9 yo female undergoing a craniotomy for tumor
PMH: medulloblastoma
PSH: none
Events: Uneventful resection. You performed a greater occipital nerve block prior to incision. During closure, the patient had a sharp decrement in her EtCO_2_ and S_p_O_2_, followed by marked hypotension. You suspect air embolism, asked the surgeon to flood the field with saline, and administer 100% F_i_O_2_. These measures failed to improve the patient’s hemodynamics. You are now beginning to suspect alternative diagnoses. When you access the cognitive aid, I’d like you to find the aid for air embolism, tell me as quickly as you can the first alternative diagnosis, and where that information can be found on the cognitive aid.

Access the cognitive aid now.
